# Yield-phenology relations and water use efficiency of maize (*Zea mays* L.) in ridge-furrow mulching system in semiarid east African Plateau

**DOI:** 10.1038/s41598-017-03372-x

**Published:** 2017-06-12

**Authors:** Fei Mo, Jian-Yong Wang, Feng-Min Li, Simon N. Nguluu, Hong-Xu Ren, Hong Zhou, Jian Zhang, Charles W. Kariuki, Patrick Gicheru, Levis Kavagi, Wesly K. Cheruiyot, You-Cai Xiong

**Affiliations:** 1 State Key Laboratory of Grassland Agro-ecosystems, Institute of Arid Agroecology, School of Life Sciences, Lanzhou University, Lanzhou, 730000 China; 2 Kenya Agricultural and Livestock Research Organization, Kabete, 14733-00800 Nairobi Kenya; 3 The Institute of Botany, Chinese Academy of Sciences, Xiangshan, Beijing 100093 China; 4 United Nations Environment Programme P.O. Box, 47074-00100 Nairobi, Kenya

## Abstract

Yield-phenology relation is a critical issue affecting rainfed maize field productivity in semiarid east African Plateau (EAP). We first introduced Chinese ridge-furrow mulching (RFM) system to EAP, using three maize cultivars with early-, mid- and late-maturing traits as test materials. A two-year field experiment was conducted in a semiarid farm of Kenya from 2012 to 2013. Three treatments were designed: alternative ridge and furrow with transparent plastic mulching (FT), with black plastic mulching (FB) and without mulching (CK). We found that FT and FB significantly increased soil moisture and accelerated crop maturity across two growing seasons. Leaf area and shoot biomass were increased by 30.2% and 67.5% in FT, 35.2% and 73.5% in FB, respectively, compared with CK. Grain yield, water use efficiency and economic output were increased by 55.6%, 57.5% and 26.7% in FT, and 50.8%, 53.3% and 19.8% in FB, respectively. Optimal yield and economic benefit were observed in late-maturing cultivar due to increased topsoil temperature in FT in 2012 (cool), and in early-maturing cultivar owing to cooling effect in FB in 2013 (warm). Our study suggested RFM system, combined with crop phenology selection, be a promising strategy to boost maize productivity and profitability in semiarid EAP.

## Introduction

Arid and semiarid areas account for more than 40% of total land area of east African Plateau (EAP), and regional climate feature is characterized by low, erratic rainfall and high evaporation^1^. This poses a very high risk of aggravating inter-annual droughts and intra-seasonal dry spells^2^. In the arid and semiarid EAP, poverty and food insecurity are intercoupling and widespread, and are closely associated with natural resource endowment such as water, thermals and soils. Inefficient rainwater collection and use, serious soil erosion and land degradation have led to low crop productivity and thus food shortages^3^. In a long run, to improve food production ability and rainwater resource use efficiency has aroused widespread concerns in semiarid EAP. However, the capacity of most east African countries to manage dryland agriculture is limited, due to widespread poverty, backwardness of dryland farming techniques, recurring droughts and unpredictable heat waves and other factors^4, 5^.

On-field rainwater harvesting technologies may provide an opportunity for increasing soil water availability and boosting crop productivity in arid and semiarid EAP^6^. Especially in smallholder-based rural areas, the technologies are expected to bring about an urgently needed increase in agricultural productivity and thus improve food and water security. In recent years, a lot of attempts have been undertaken to examine the efficiency and suitability of traditional and *in situ* rainwater harvesting technologies in EAP. For instance, terraced fields are to some extent established to increase soil water storage and crop yield in semiarid areas of Kenya^7^. Other rain-harvesting strategies are regionally applied in EAP, such as pitting cultivation system in Ethiopia and stone bund establishment in Tanzania^8, 9^. Also, straw mulching was widely used to restrain soil evaporation and improve infiltration ability in sloping land^10^. In spite of this, these indigenous technologies did not achieve enough environmental and economic effects as desired in semiarid EAP. For one thing, most of technologies only showed short-term effects on soil water conservation under intensive soil evaporation and uncertain rainfall^11^. For another, inappropriate use of these technologies and insufficient participation by farmers further lead to failed adoption processes and weaker-than-expected effects^9, 12, 13^.

Facing such problems aforementioned, an innovative *in situ* rainwater harvesting system, i.e. ridge-furrow mulching system (RFMS), has been established and extensively used in dryland agriculture over last two decades in semiarid Loess Plateau (LP) of China^14^. The RFMS is comprised of alternative ride-furrow units and soil surface mulching with different materials^15^. The ridges and furrows are used to collect and store rainwater^16^. The mulching materials, typically including plastic film (black or transparent films), gravel sand, crop/grass straw and others^17^, serve as the media to prevent soil water evaporation and moderate thermal balance. Either plastic film or straw represents the widely used material, especially in the areas with insufficient rainfall and poor soil. By virtue of its multiple advantages of low cost and simple operation, the RFMS is easily adopted by local smallholder farmers in the rainfed agricultural area of northwest China^18^. Since the early 2000s, RFMS has been extensively used in maize production in semiarid China^18–22^, owing to its high efficiency in rainwater collection^23^, soil water conservation and field productivity improvement^24^. The extension area has reached up to 840,000 ha in Gansu province only (northwest China) in 2012, which accounted for 24% of local total arable lands. Large area application of RFMS has already contributed to more than 50% of local total grain production since then.

Till now, there were little previous studies to address the agronomic and physiological roles of crop phenology under RFMS. Crop phenological adaptation is frequently associated with its inherent maturity traits^25^. Maturity group determinates crop phenology pattern, and is therefore one of the important growth traits for determining crop yield on a specific location with local climate condition^25^. Within a growing season, it has usually been observed that the production of late-maturing cultivars is greater than that of early-maturing cultivars. This is mainly due to the fact that late-maturing varieties have a longer reproductive period and can take full advantage of the water and thermal resources in growing season^26^. However, late-maturing cultivar adoption could bring a certain risk when the crops are subjected to some unexpected environmental stresses during critical growth stages. The most outstanding risk in EAP is that crop could be damaged by heat and drought stresses at the late stage of growing season, and harvest lesser yield and quality^27^. Clearly, facing different climatic conditions, optimizing genotype selection on maturity group plays an important role in improving crop yield. Moreover, the RFMS has been deemed as a useful farming practice to increase soil water availability, moderate thermal balance and hence prolong the duration of desired growth stage. Yet, it is unclear how the coupling relationship between RFMS and phenological selection would be, in terms of crop yield output and water use efficiency in EAP.

In this study, we first introduced Chinese RFMS to EAP, with the aim to reveal yield-phenology relation and its physiological role in upgrading local farming system. The alternative ridge-furrow unites with two different mulching materials (transparent and black plastic films) were investigated in two growing seasons. According to our previous observations, transparent film help increase the temperature of topsoil while black one turned to decrease it^16^. Considering local rainfall patterns and thermal conditions, three maize cultivars with contrasting maturity groups, i.e. early-, mid- and late-maturing cultivars, were tested and compared in RFMS. The primary objectives of this study were: 1) to evaluate the overall effectiveness of introduced RFMS technology on growth, grain yield and water use efficiency in cool and warm growing seasons, 2) to compare the differences in grain yield and economic benefits among three cultivars with contrasting maturity traits, and 3) to identify the optimum combinations between cultivar phenological traits and mulching materials in cool and warm growing seasons in EAP.

## Results

### Rainfall and temperature during growing season

**Figure 1:**
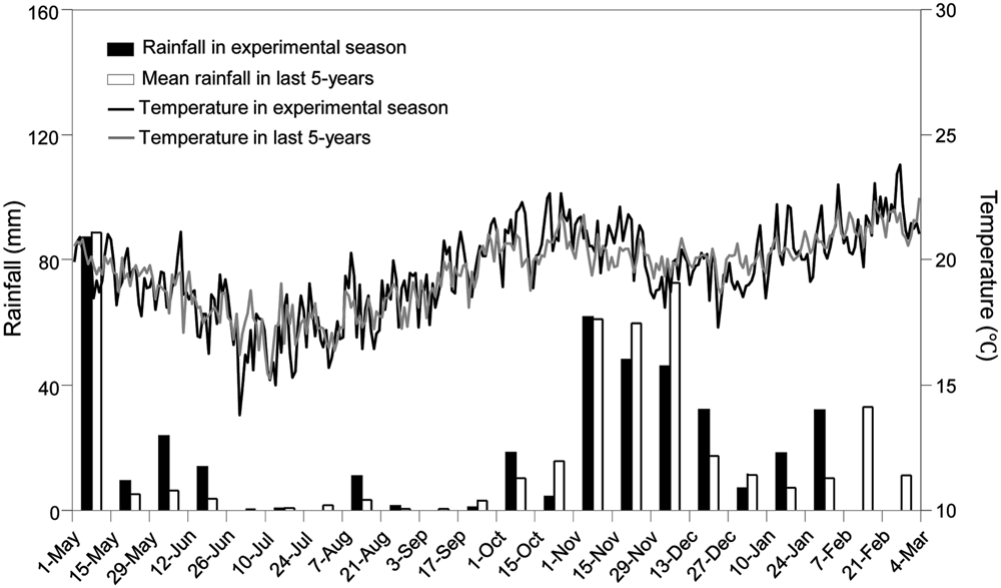
Biweekly precipitation distributions and daily mean air temperature in the experimental seasons and last 5 years (2009‒2013) in KARI-Katumani Centre, Kenya.

The 2012 growing season, known as a cool and long rainy season, was characterized with relatively low air temperature (18.5 °C), total rainfall amount of 127.3 mm from April to July. The 2013 growing season was a relatively warm and short rainy season, with relatively high air temperature of 20.6 °C, and rainfall amount of about 189.6 mm from late-November to next mid-February (Fig. 1).

### Comparisons of soil water storage at 0‒120 cm depth

In 2012 growing season, mulching significantly affected soil water storage (SWS) at vegetative stages (i.e. seedling and jointing), but rather than at reproductive stages including heading, filling and maturity (Table 1). Regardless of maize cultivar, there was significantly higher SWS in FT and FB than CK (*p* < 0.05), while there was no significant difference in SWS between FT and FB. On the other hand, cultivar genotypes significantly affected SWS at the late growth stages such as jointing, heading and filling stage. In general, early-maturing cultivar KDVI achieved greater water storage amount than mid- and late-maturing ones did. When the data was subjected to two-way ANOVA, the combination between crop genotypes and mulching treatments significantly affected the dynamics of SWS at the jointing stage.

**Table 1:** Soil water storage (0–120 cm soil layer) as affected by maize cultivars (early- mid- and late-maturing, KDVI, JK3 and FN1, respectively) and ridge-furrow differingin mulching materials (FT, alternative ridge-furrow with transparent film mulching; FB, alternative ridge-furrow with black film mulching and CK, alternative ridge-furrow with no mulching) in both growing seasons, on a sandy clay loam in Kenya, east African Plateau.

Season	Cultivar	Mulching	Soil water storage (mm)				
			Seedling	Jointing	Heading	Filling	Maturity
2012	KDVI	FT	224.8a	248.1a	193.0a	174.9a	160.5a
		FB	215.1a	238.6a	187.6a	186.8a	152.3a
		CK	178.3b	193.2b	179.9a	170.9a	132.7b
	JK3	FT	212.1a	228.9a	160.8a	164.9a	150.7a
		FB	225.9a	234.8a	188.1b	173.3a	166.7a
		CK	180.3b	206.9b	164.0a	162.7a	150.4a
	FN1	FT	214.2a	227.8a	169.9a	163.3a	139.4a
		FB	216.6a	236.6a	162.4a	148.9b	155.6a
		CK	178.3b	181.3b	163.9a	147.4b	158.4a
	ANOVA results	Cultivar (C)	ns	**	**	***	ns
		Mulching (M)	***	***	ns	ns	ns
		C × M	ns	**	ns	ns	ns
2013	KDVI	FT	236.3a	269.8a	193.8a	168.0a	147.2a
		FB	250.4b	263.0a	194.5a	174.4a	148.2a
		CK	192.0c	203.5b	171.3b	153.7a	140.1b
	JK3	FT	226.3a	255.8a	176.4a	169.4a	144.7a
		FB	229.7a	253.9a	180.3a	165.6a	142.0a
		CK	194.0b	195.2b	146.5b	145.8b	137.0a
	FN1	FT	236.3a	238.5a	167.7a	168.5a	148.2a
		FB	233.8a	244.7a	159.2a	168.6a	143.7a
		CK	191.7b	198.7b	137.2b	146.9b	137.4b
	ANOVA results	Cultivar (C)	ns	**	***	ns	ns
		Mulching (M)	***	***	***	***	*
		C × M	ns	ns	ns	ns	ns

Values followed by the same letters in each cultivar are not significantly different at *p* < 0.05 level. *, ** and ***significant at *p* < 0.05, 0.01 and 0.001 level, respectively; ns, not significant.

In 2013 growing season, the effects of mulching treatments on SWS were truly significant across whole growing period (Table 1). Across all three cultivars, FT and FB achieved significantly (*p* < 0.05) greater water storage amount, while in most cases there was no significant difference in SWS between FT and FB. As similar as in 2012 growing season, cultivar genotypes also played significant effects on water storage in soil at jointing and heading stages. Irrespective of mulching treatments, SWS was 10.4 mm and 18.2 mm greater at jointing stage, and 18.8 mm and 31.9 mm greater at heading stage in early-maturing KDVI than mid-maturing JK3 and late-maturing FN1, respectively.

### Differentiate performance of topsoil temperature at 10 cm depth

**Figure 2:**
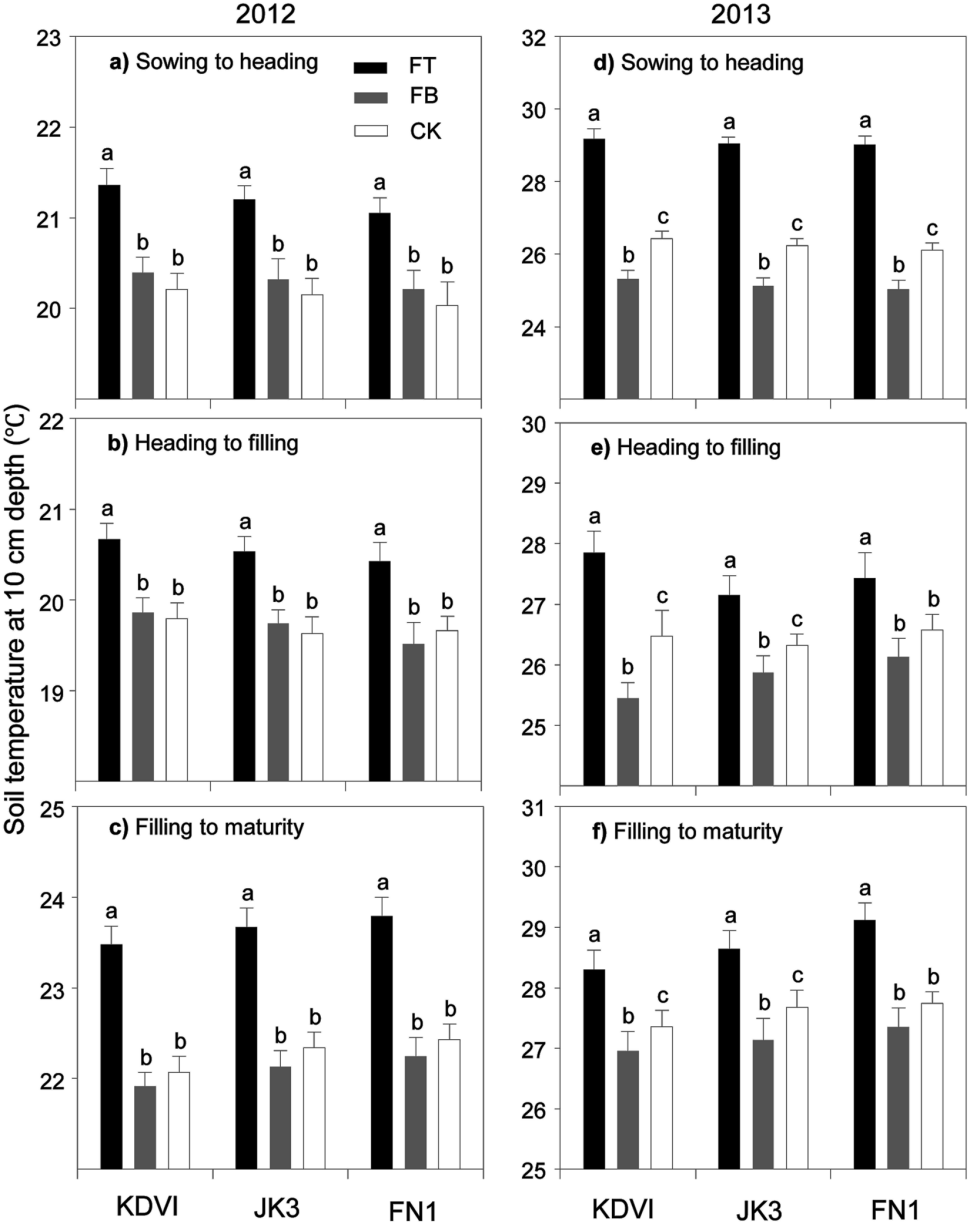
Soil temperature (10 cm soil depth) as affected by maize cultivars (early- mid- and late-maturing, KDVI, JK3 and FN1, respectively) and ridge-furrow differing in mulching materials (FT, alternative ridge-furrow with transparent film mulching; FB, alternative ridge-furrow with black film mulching and CK, alternative ridge-furrow with no mulching) in both growing seasons, on a sandy clay loam in Kenya, east African Plateau. Values followed by the same letters in each cultivar are not significantly different at *p* < 0.05 level. *, ** and ***significant at *p* < 0.05, 0.01 and 0.001 level, respectively; ns, not significant.

The changes in mean soil temperature at three key developmental stages (sowing to heading, heading to filling and filling to maturity) were presented in Fig. 2. In comparison with FB and CK, FT significantly increased topsoil temperature in all cultivars across two growing seasons. Furthermore, FB shared similar time-course dynamics in soil temperature as CK did in 2012 growing season. When it came to 2013 growing season, however, FB significantly (*p* < 0.05) reduced soil temperature by 1.1 °C from sowing to heading, 0.64 °C from heading to filling and 0.45 °C from filling to maturity, respectively, as compared with CK. The differences in soil temperature among the cultivars were not significant over two growing seasons. FB treatment recorded greater soil temperature reduction in the early-maturing KDVI (1.03 °C) plots than that of mid-maturing JK3 (0.45 °C) and late-maturing FN1 plots (−0.44 °C).

### Phenological traits of contrasting cultivars under RFMS

As expected, the length of each phenophase was shorter in early-maturing KDVI than that of mid-maturing JK3 or late-maturing FN1 across two growing seasons (Table 2), which was closely associated with crops’ maturity groups. Generally, mulching treatments and cultivar genotypes significantly altered the duration of each main growth period over two growing seasons. The length of vegetative growth (i.e. from sowing to heading) was shorter in FT than FB across three cultivars over two growing seasons. The longest vegetative growth was observed in CK. Specifically, in FT treatment, the mid- and late-maturing cultivars displayed greater reduction in the period from sowing to heading, compared with early-maturing KDVI. Furthermore, the period from heading to filling was longer in late-maturing FN1 than early- and mid-maturing cultivars over two growing seasons. Particularly in 2013 growing season, this period was prolonged in cultivar JK3 and FN1 but not in early-maturing KDVI. On the other hand, the FT and FB treatments resulted in significantly longer duration of grain filling than CK did in two growing seasons, while in most cases there were no significant differences in phenological stages between FT and FB in all cultivars. As a result, FT and FB treatments significantly shortened the length of growing season in comparison with CK, irrespective of cultivar types.

**Table 2:** Phenology as affected by maize cultivars (early- mid- and late-maturing, KDVI, JK3 and FN1, respectively) and ridge-furrow differingin mulching materials (FT, alternative ridge-furrow with transparent film mulching; FB, alternative ridge-furrow with black film mulching and CK, alternative ridge-furrow with no mulching) in both growing seasons, on a sandy clay loam in Kenya, east African Plateau.

Season	Cultivar	Mulching	Phenology (days)				
			Sowing‒ jointing	Jointing‒ heading	Heading‒ filling	Filling‒ maturing	Sowing‒ maturity
2012	KDVI	FT	38a	57a	11a	22a	93a
		FB	39a	62b	11a	21a	94a
		CK	44b	69c	15b	17b	99b
	JK3	FT	42a	67a	13a	26a	105a
		FB	43a	71b	13a	23b	105a
		CK	48b	78c	16b	18c	110b
	FN1	FT	45a	70a	14a	26a	109a
		FB	46a	76b	15a	24a	112b
		CK	50b	84c	16a	17b	116c
	ANOVA	Cultivar (C)	***	***	***	*	***
		Mulching (M)	***	***	***	***	***
		C × M	ns	ns	ns	ns	ns
2013	KDVI	FT	32a	49a	13a	26a	89a
		FB	31a	50a	14a	24a	89a
		CK	36b	60b	15a	20b	95b
	JK3	FT	37a	56a	18a	22a	97a
		FB	36a	56a	18a	23a	96a
		CK	43b	69b	24b	16b	105b
	FN1	FT	37a	58a	20a	23a	101a
		FB	37a	59a	20a	24a	102a
		CK	44b	72b	25b	17b	112b
	ANOVA	Cultivar (C)	***	***	***	**	***
		Mulching (M)	***	***	***	***	***
		C × M	ns	ns	ns	ns	*

Values followed by the same letters in each cultivar are not significantly different at *p < *0.05 level. *, ** and ***significant at *p < *0.05, 0.01 and 0.001 level, respectively; ns, not significant.

### Leaf area and above-ground biomass at heading stage

**Figure 3:**
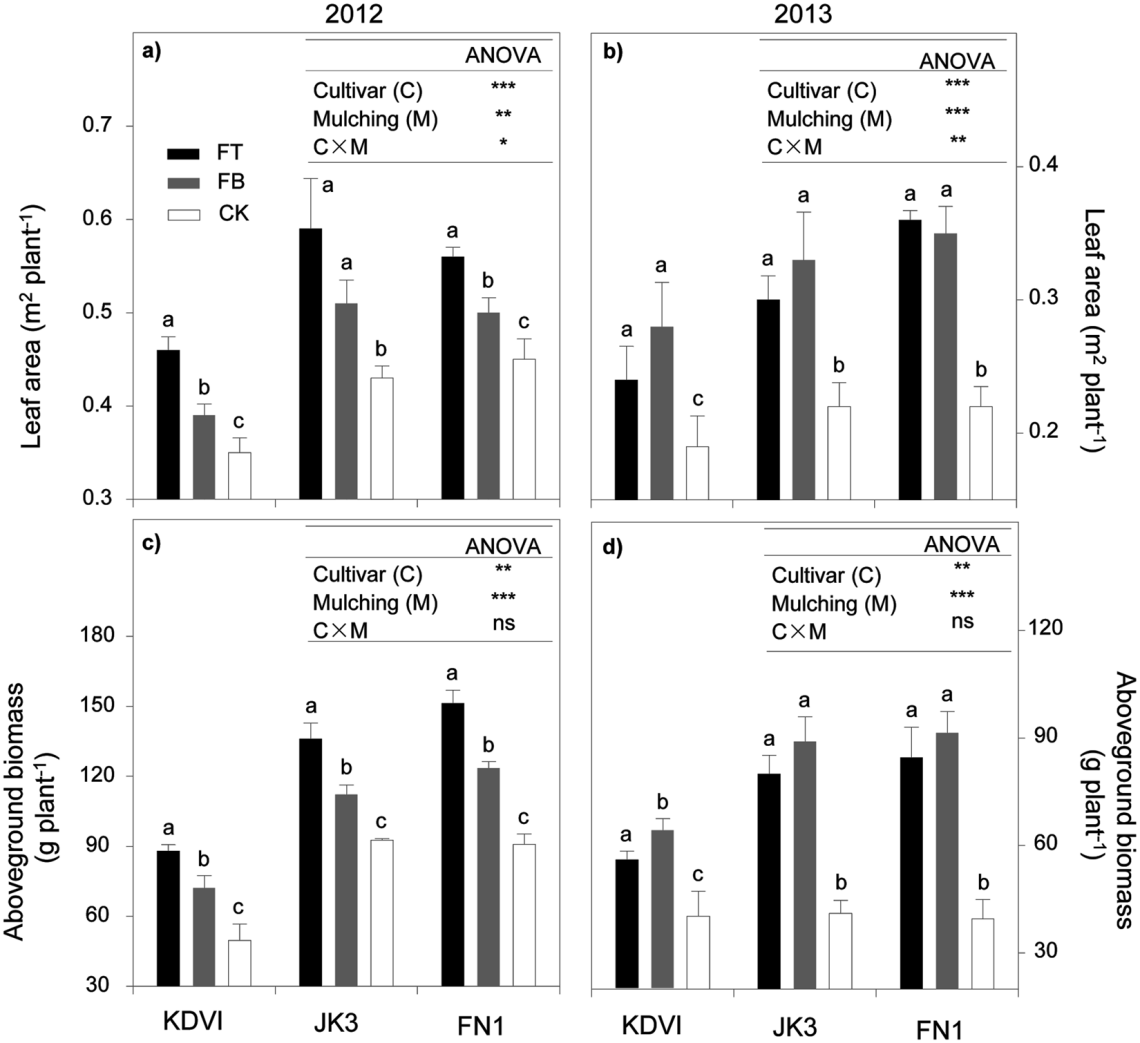
Leaf area and aboveground biomass as affected by maize cultivars (early- mid- and late-maturing, KDVI, JK3 and FN1, respectively) and ridge-furrow differing in mulching materials (FT, alternative ridge-furrow with transparent film mulching; FB, alternative ridge-furrow with black film mulching and CK, alternative ridge-furrow with no mulching) in both growing seasons, on a sandy clay loam in Kenya, east African Plateau. Values followed by the same letters in each cultivar are not significantly different at *p* < 0.05 level. *, ** and ***significant at *p* < 0.05, 0.01 and 0.001 level, respectively; ns, not significant.

Leaf area was significantly affected by either crop genotypes or mulching treatments in two growing seasons (Fig. 3a and b). In general, average leaf area across three cultivars was increased by 44.4% and 18.5% in FT and FB in 2012, 38.0% and 51.5% in FT and FB in 2013, respectively. For cultivar JK3 and FN1, leaf area was increased by 62.7% and 74.4% in 2012, and 31.2% and 34.7% in 2013, respectively. On the other hand, the interaction between cultivar and mulching significantly affected above-ground biomass accumulation in both seasons (Fig. 3c and d). The greatest biomass was observed in FT treatment in 2012, and in FB treatment in 2013. Compared with CK, FT and FB significantly improved biomass production, in an average of three cultivars, by 54.0% and 38.8% in 2012, and 82.1% and 102.1% in 2013, respectively. Regardless of mulching materials, late-maturing FN1 achieved the greatest above-ground biomass over two growing seasons.

### Yield, yield component and water use efficiency (WUE)

**Figure 4:**
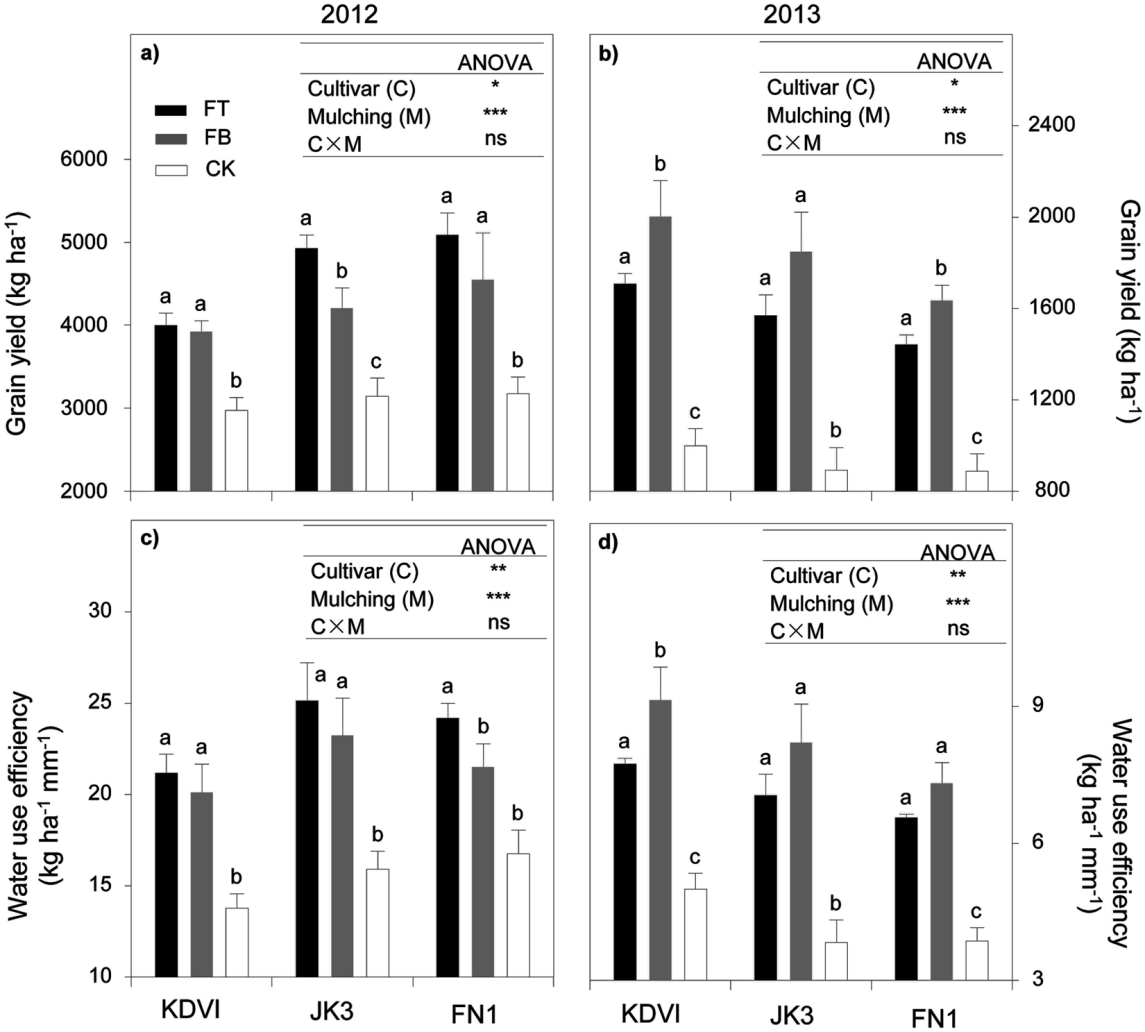
Grain yield and water use efficiency as affected by maize cultivars (early- mid- and late-maturing, KDVI, JK3 and FN1, respectively) and ridge-furrow differing in mulching materials (FT, alternative ridge-furrow with transparent film mulching; FB, alternative ridge-furrow with black film mulching and CK, alternative ridge-furrow with no mulching) in both growing seasons, on a sandy clay loam in Kenya, east African Plateau. Values followed by the same letters in each cultivar are not significantly different at *p* < 0.05 level. *, ** and ***significant at *p* < 0.05, 0.01 and 0.001 level, respectively; ns, not significant.

Yield-related components were different from the treatments and crop genotypes. In 2012 growing season, cultivar types and mulching treatments accounted for the differences in all yield-related parameters as indicated in Table 3. In general, early-maturing cultivar KDVI appeared to have significantly lower values of all yield components than the mid- and late-maturing cultivars did. For each cultivar, FT and FB totally increased the values of these components significantly, whereas there were no significant differences in most of yield component parameters between FT and FB, except for kernel abortion rate, kernel weight per ear and thousand-grain weight. As a result of yield components improvement, grain yield and WUE were totally increased, in average across three cultivars, by 50.7% and 51.8% in FT, and 36.3% and 39.6% in FB, respectively (Fig. 4a and c). With regards to crop genotypes, late-maturing cultivar FN1 showed massive increases in grain yield and WUE averagely by 17.6% and 16.7%, respectively, compared with early-maturing cultivar KDVI.

**Table 3:** Yield components as affected by maize cultivars (early- mid- and late-maturing, KDVI, JK3 and FN1, respectively) and ridge-furrow differingin mulching materials (FT, alternative ridge-furrow with transparent film mulching; FB, alternative ridge-furrow with black film mulching and CK, alternative ridge-furrow with no mulching) in both growing seasons, on a sandy clay loam in Kenya, east African Plateau.

Season	Cultivar	Mulching	Yield component					
			Ear length (cm)	Ear diameter (cm)	Kernel abortion (%)	Kernel number per ear	Kernel weight per ear (g)	1000-grain weight (g)
2012	KDVI	FT	15.7a	4.6a	23.2%a	470.4a	77.3a	239.0a
		FB	15.4a	4.5a	23.0%a	469.1a	78.4a	240.4a
		CK	13.5b	4.3b	27.5%b	407.4b	58.2b	204.9b
	JK3	FT	17.0a	5.2a	28.5%a	608.8a	98.5a	245.8a
		FB	16.4a	5.1a	34.1%b	613.4a	77.5b	217.6b
		CK	15.3b	4.7b	37.7%c	578.2b	60.2c	183.3c
	FN1	FT	18.1a	5.3a	28.5%a	617.4a	101.7a	251.9a
		FB	17.8a	5.1a	31.9%b	597.6a	91.0b	224.1b
		CK	15.3b	4.8a	39.7%c	527.7b	61.5c	192.5c
	ANOVA	Cultivar (C)	***	***	***	***	*	**
		Mulching (M)	***	***	**	**	***	***
		C × M	ns	ns	ns	ns	ns	**
2013	KDVI	FT	13.1	3.8	41.5%	414.0	33.5	141.0
		FB	14.8	4.2	40.9%	492.9	40.7	155.3
		CK	11.2	3.6	36.1%	404.3	27.1	136.3
	JK3	FT	13.3	4.3	54.2%	471.3	32.1	139.5
		FB	14.1	4.5	47.3%	494.9	37.6	126.7
		CK	12.2	4.1	43.1%	436.2	22.3	127.0
	FN1	FT	12.7	4.0	44.1%	493.2	30.8	121.7
		FB	14.0	4.2	41.5%	526.9	35.3	136.4
		CK	11.3	3.4	46.2%	467.5	23.4	83.8
	ANOVA	Cultivar (C)	ns	***	ns	***	ns	***
		Mulching (M)	**	***	ns	***	***	***
		C × M	ns	ns	ns	ns	ns	**

Values followed by the same letters in each cultivar are not significantly different at *p* < 0.05 level. *, ** and ***significant at *p* < 0.05, 0.01 and 0.001 level, respectively; ns, not significant.

In 2013 growing season, mulching treatment significantly changed the values of most yield components, except for kernel abortion rate (Table 3). In most cases, FB generally registered the greater values of components than FT did. The values of all yield components in CK were generally the lowest across all cultivars. Crop genotypes accounted for significant effects on yield components such as ear diameter, kernel number per ear and thousand-grain weight. Specifically, mid- and late-maturing cultivars tended to obtain greater ear diameter and kernel number per ear, while early-maturing cultivar KDVI maintained greater kernel weight per ear. Consequently, both grain yield and WUE in FB treatment were significantly improved by 100.1% and 106.0%, respectively, comparing with CK (Fig. 4b and d). Finally, early-maturing cultivar KDVI was observed to have significantly greater grain yield and WUE by 19.2% and 20.3% than late-maturing cultivar FN1 did.

### Economic benefits

In this study, we also made an econometric analysis on the output to input ratio regarding RFM treatments and crop genotypes. According to local labour price level, total labour input was 72.2 and 70.6 US$ ha^−1^ higher in mulching groups (i.e. FB and FT, labour input of FB was same as that of FT) than that of CK in 2012 and 2013 growing seasons, respectively (Table 4). In two growing seasons, an extra input (194.8 US$ ha^−1^) of commercial plastic film was involved in FB and FT groups. As a result, total input was 267.0 and 265.4 US$ ha^−1^ higher in plastic film-mulching groups than that of CK in 2012 and 2013 growing seasons, respectively. Regarding the output in 2012 (long rainy season), the highest value was found in FT group and the lowest one in CK for each cultivar. Consequently, the highest net economic income was 1236.7US$ ha^−1^ in late-maturing cultivar FN1 in FT. For the output in 2013 (short rainy season), FB group registered the maximum economic benefits across all cultivars, and the difference in economic benefit between FT and CK was not statistically significant. The early-maturing cultivar, in combination with FB, harvested the greatest net income, up to 209.8 US$ ha^−1^ in this season.

**Table 4:** Economic benefits as affected by maize cultivars (early- mid- and late-maturing, KDVI, JK3 and FN1, respectively) and ridge-furrow differingin mulching materials (FT, alternative ridge-furrow with transparent film mulching; FB, alternative ridge-furrow with black film mulching and CK, alternative ridge-furrow with no mulching) in both growing seasons, on a sandy clay loam in Kenya, east African Plateau.

Season	Cultivar	Mulching	Input values of consumable items (US$ ha^−1^)				Output values of crop yield (US$ ha^−1^)				Net income (US$ ha^−1^)
			Labor	Seed	Plastic film	Total	Straw	Grain	Total		
2012	KDVI	FT	297.5	13.9	194.8	506.2	103.6	1199.6	1303.2		797.0
		FB	297.5	13.9	194.8	506.2	126.6	1175.3	1301.9		795.7
		CK	225.3	13.9	0	239.2	71.4	892.8	964.2		725.0
	JK3	FT	297.5	13.9	194.8	506.2	195.9	1478.2	1674.1		1167.9
		FB	297.5	13.9	194.8	506.2	161.6	1262.0	1423.6		917.4
		CK	225.3	13.9	0	239.2	133.4	943.6	1077.0		837.8
	FN1	FT	297.5	13.9	194.8	506.2	217.6	1525.3	1742.9		1236.7
		FB	297.5	13.9	194.8	506.2	177.7	1364.6	1542.3		1036.1
		CK	225.3	13.9	0	239.2	130.9	953.2	1084.1		844.9
2013	KDVI	FT	274.3	13.9	194.8	483.0	80.2	512.2	592.5		109.5
		FB	274.3	13.9	194.8	483.0	92.1	600.7	692.8		209.8
		CK	203.7	13.9	0	217.6	58.0	266.6	354.7		137.1
	JK3	FT	274.3	13.9	194.8	483.0	115.0	471.0	586.0		103.0
		FB	274.3	13.9	194.8	483.0	128.0	554.4	682.4		199.4
		CK	203.7	13.9	0	217.6	59.1	235.0	324.0		106.4
	FN1	FT	274.3	13.9	194.8	483.0	121.6	432.3	554.0		71.0
		FB	274.3	13.9	194.8	483.0	131.7	489.9	621.6		138.6
		CK	203.7	13.9	0	217.6	56.9	230.5	317.5		99.9

Price per unit for grain: 0.3 US$ kg^−1^, for dry straw: 0.03 US$ kg^−1^.

## Discussion

Improving food production ability and rainwater resource use efficiency has aroused widespread concerns in semiarid east African Plateau (EAP). We first introduced Chinese micro-field rain-harvesting farming system to EAP, in combination with genotype selection with contrasting maturity groups. The RFMS can increase rainwater collection and infiltration through collecting runoff from ridges and conserving water in furrows^28^, reducing evaporation and increasing soil moisture^22^. Previous studies have showed that the RFMS was efficient in improving soil water availability in semiarid northwest China^23^. Our data indicated that SWS amount in plastic-mulching treatments was significantly higher than that of CK, and the differences in SWS between them was massive at the vegetative stages rather than at reproductive stages (late growth stages) (Table 1). This may mechanically be explained by temporal distribution of rainfall and crop water demand during growing season. In our study site, rainfall mainly occurred at early stages of growing season. Yet, the differences in soil water status among treatments turned to become smaller at late developmental stages. These results were in good agreement with the phenomenon observed in the semiarid northwest of China^18, 29^. Additionally, it is widely accepted that early-maturing cultivars usually consume less water owing to their shorter growth duration and less biomass accumulation^30^. Our study also found that irrespective of mulching materials, total SWS amount was significantly higher in early-maturing KDVI than that of mid- or late-maturing cultivar group at the jointing and heading stages over two growing seasons.

It is well known that soil surface mulching with polyethylene film helps increase soil temperature and suppress thermal loss through evaporation^15^. The warming effect is mostly marked under transparent film mulching, and black film mulching is not as effective as transparent film mulching in raising the temperatures due to low penetration for sunlight^31^. To date, transparent film mulching has been widely used to improve thermal condition in many cool and arid regions in the Loess Plateau. For instance, Wang *et al*. found that in early growing season, topsoil temperature in the film mulched plots was 3.0–6.8 °C higher than that of the uncovered one, while the difference tended to be smaller in late growing season^32^, probably due to canopy closure that intercepted the majority of solar energy and thus minimized the effects caused by mulching. Similar phenomenon was observed by Jia *et al*. in alfalfa in semiarid Loess Plateau^33^. Our observations affirmed the warming effects of transparent film on topsoil layer. Nevertheless, in this study, topsoil temperature in the black film mulching group showed no-significant difference in comparison with that of the control in 2012 growing season, while the former turned to be significantly lower than the latter in 2013 growing season (Fig. 2). Ai *et al*. also observed that the coverage with black film, as blocking the passage of most solar radiation, slightly reduced soil temperature, comparing with the unmulched treatment^34^. In our study site, local mean annual temperature was up to 19.2 °C. Heat stress frequently took place in most time of growing season, which played a negative role in crop growth and grain filling. In this case, the cooling effect of black film on topsoil layer appeared to be beneficial to crop growth and reproduction, especially in late stage of 2013 growing season.

The influences of crop phenology characteristics on leaf area, biomass accumulation and final yield have been widely investigated in crop production. Existing studies showed that the improved soil temperature and moisture due to film mulching usually accelerated crop growth^35, 36^ and increased leaf area and above-ground biomass^20, 37^. We found that comparing with the unmulched control, the durations from sowing to jointing and from jointing to heading were the shortest in transparent film mulching group, followed by black film group. Li *et al*. also observed that maize seedlings grown in plastic-covered plots emerged 4–15 days earlier than unmulched controls^38^. Similarly, potato mulched with plastic film emerged 12 days earlier than those of unmulched controls^39^. Interestingly, we found that the advanced vegetative phenophases did not shorten the length of reproductive growth, especially the grain filling duration. Our data indicated that the length from filling to maturity was prolonged in two film mulching treatments in comparison with that of unmulched control (Table 2). This result was in agreement with the finding by Bu *et al*. who reported that the heading stage of maize was 17 days earlier in the film mulching treatment than that the unmulched control^20^. Furthermore, film mulching extended the duration of reproduction (i.e. heading to maturing) by 11 days, in comparison with CK. In addition to crop phenology, previous studies have demonstrated that film mulching remarkedly increased leaf area by maintaining relatively fine heat and water conditions during vegetative stages^37, 40–42^. Increased radiation capture due to higher LAI resulted in greater biomass accumulation^43^. In this study, the greatest leaf area and above-ground biomass were obtained in the transplant film mulching treatment in 2012 growing season, and in the black film treatment in 2013 growing season, respectively (Fig. 3). This phenomenon was mostly associated with improved thermal balance. As mentioned above, 2012 growing season was relatively cool, and transparent film mulching can help improve soil temperature and hence promote crop growth. In contrast, 2013 growing season was relatively warm, and the cooling effect under black film mulching was beneficial for biomass accumulation and leaf development.

However, the enhancement of crop yield is not solely related to the increase in sources (leaf area, biomass accumulation) but also the improvement in yield components such as size of ear, number and weight of kernel etc. Mounting evidence indicated that film mulching significantly improved yield formation in many crops such as maize^18, 21, 44^, potato^29, 37^ and wheat^45^. Our data indicated that in 2012 growing season, the significant increases in ear size, number and size of kernel eventually led to higher grain yield under the condition of transparent film mulching. Nevertheless, it was not true in 2013 growing season where black film maintained best performance in yield components and hence grain yield (Table 3 and Fig. 4a and b). As aforementioned, the crops were frequently subjected to drought and heat stresses during the reproduction stage in 2013 growing season. High temperature and drought at flowering stage usually led to the loss of pollen activity^46^ and the reduction of pollination chance by prolonging the interval period from anthesis to silking^47^. Our data also indicated that the interval time from heading to filling was prolonged and the kernel abortion was greatly higher in 2013 than2012 (Table 3). As a result, grain yield was in average 3-fold lower in 2013 than that of 2012 (Fig. 4a and b). However, the use of black film not only increased soil water storage but also slightly decreased soil temperature in 2013, therefore leading to greater grain yield. This indicated that black film mulching in warm season could serves as a compensation for yield losses induced by heat and drought stresses. Also, our data registered the genotypic difference in grain yield between two growing season. The greatest yield was obtained in thelate-maturing cultivar in 2012 growing season, and in the early-maturing cultivar in 2013 growing season. This was possibly because the early-maturing cultivar could avoid serve drought and heat stresses occurred in the latter part of growing season, through flowering and grain filling earlier^48^, and tended to produce more stable yield output. However, in a season of relatively favourable conditions, the late-maturing cultivar was more likely to produce more biomass and thus greater yield potential than early-maturing one^30^. In conclusion, the greatest grain yield and economic harvest were achieved by late-maturing cultivar in FT in 2012 growing season (cool) as a result of increased topsoil temperature under transparent film mulching (mainly at early growing stage), and by early-maturing cultivar in FB in 2013 growing season (warm) due to the cooling effect under black film mulching (mainly at late growing stage).

**Figure 5:**
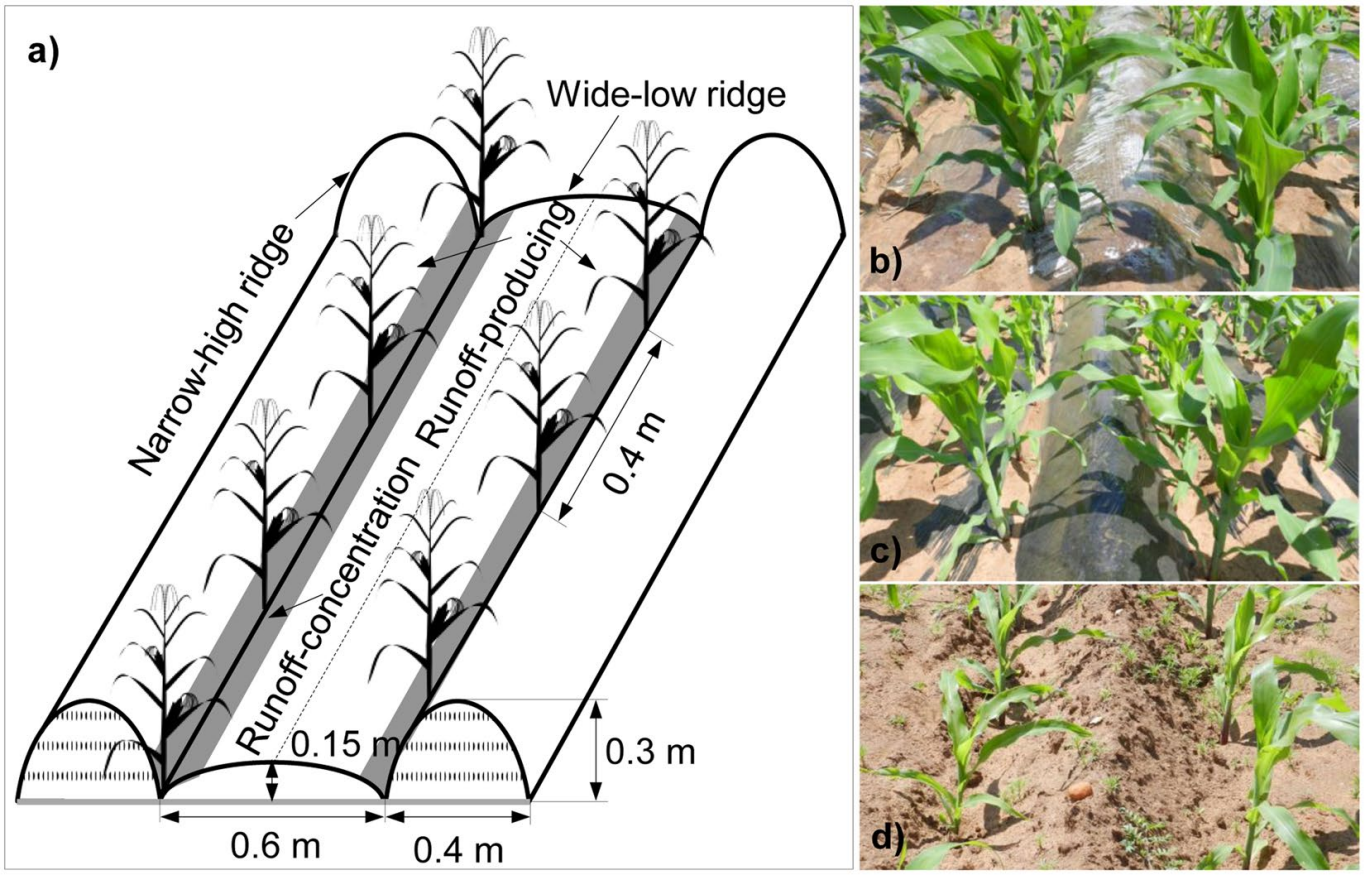
The schematic diagram of alternative ridge-furrow unit (**a**) and different mulching materials (**b**,**c** and **d**) in both experimental seasons.

In addition to grain yield, ridge-furrow planting with film mulching significantly increased WUE in many crop plants^33, 42, 45, 49^. We observed that compared with CK, both transparent and black film mulching treatments increased WUE by 51.6% and 39.4% in 2012, and by 42.0% and 64.0% in 2013, respectively (Fig 5c and d). The regulatory mechanisms behind it were most likely attributable to, 1) increase crop yield due to accelerated plant growth, and ultimately lead to reproductive success^15^, 2) promote water availability at critical stages of crop water demand and 3) increase leaf area and biomass accumulation due to improved soil moisture and thermal conditions. With the improvements of grain yield and WUE, net economic income was accordingly increased under film mulching conditions^18, 37^. Therefore, the use of ridge and furrow with film mulching in maize production may serve as a viable tool to reduce production risks associated with extreme climate and to improve farmers’ livelihoods in semiarid areas of EAP.

Local farming system is dominated by flat planting in semiarid rainfed agricultural area of east African Plateau ﻿(Table S1). Some macro-catchment rainwater harvesting technologies such as tomb-style, geosyncline-style and trench-style catchments are used only in very limited areas^8^. Due to the restrictions of multiple factors such as terrain conditions, engineering investment and insufficient socioeconomic capability, the extension area and yield-increasing contribution of macro-catchment technologies are largely limited^9^. In the meantime, micro-field rain-harvesting cultivation technologies are rarely used. The farming technologies tested in this study not only overcame the obstacle of natural rainwater collection, but also facilitated the rainwater storage within growing season. Importantly, the technologies had the advantages of simple operation, low cost and efficient output, and can be widely recognized by local farmers. We had also conducted technology demonstration and application at farmer household scale in Juja, Machakos and other counties of Kenya, which was widely adopted by local farmers. For example, in a village around Juja County (10 km northwest to Juja), we completed the technology application at a household farmland (almost 0.3 hectare), and received the positive recognition from all the villagers. In recent four years, we had organized a few field training courses for local farmers and technicians in typical sites of Kenya, and there have been over ten thousand visitors or trainees to participate into our training courses. In this study, we also attempted testing the phenological adaptability of maize varieties with different maturity group, in terms of the context of local farming system. Actually, local maize cultivars belonged to early-maturing genotypes (short growth cycle) with low yield potential. Particularly in long rainy season, it was difficult for early-maturing cultivars to make full use of water and heat resources in growing season, due to the limited length of growing period. This practice frequently resulted in a low output level and water use efficiency. In this case, we introduced the cultivars with long growing period to Kenya, with the aim to fully utilize the improved water and heat conditions under ridge-furrow mulching system. The data indicated that the introduced farming system (crop cultivars + farming technologies) achieved pronounced effects on improving rainwater use efficiency and maize yield at farmer household scale in semiarid Kenya. To sum up, our efforts had exerted important application value for local farming system and farmers in Kenya.

## Conclusions

The introduced on-field rainwater harvesting practices, alternative ridge-furrow with film mulching, significantly increased maize yield, water use efficiency and thus economic benefits compared with conventional planting pattern. The reasons behind these differences can be assigned to the fact that improved soil moisture and temperature conditions accelerated crop vegetative development, enhanced the leaf area and promoted the accumulation of above-ground biomass under RFM conditions, ultimately leading to the optimized yield components and reproduction distribution. This study also identified the optimum combinations between mulching materials and crop genotypes over two growing seasons. Specifically, the application of ridge-furrow farming with transparent film mulching using the late-maturing cultivar achieved the optimal productivity and net income in cool growing season. In contrast, the early-maturing cultivar in ridge-furrow system with blank film mulching obtained the greatest grain yield and economic benefit in warm growing season. For the first time, we identified that yield-phenology relation help upgrade this farming system. In conclusion, the introduction of alternative ridge-furrow film mulching should be served as a promising beneficial practice to improve rainwater harvesting, maize productivity, economic benefits and hence farmers’ livelihood in semiarid areas of EAP. However, the efficacy and stability of these optimum combinations still would require further field validation in the region.

## Materials and Methods

### Description of experimental site

The experimental field was established at the Kenya Agriculture Research Institute-Katumani Centre (1°35´S, 37°14´E), south-eastern Kenya from 2012 to 2013. The experimental site belongs to typical semiarid climate, with an elevation of 1600 m, mean annual rainfall of 655 mm and mean annual temperature of 19.2 °C. Local annual potential evaporation amount is up to 1840 mm. Dryland maize in this area is usually cultivated in two rainy seasons. The soil is sandy clay loam in texture and chromic luvisols in classification^50^, with average bulk density of 1.48 g cm^−3^. Local field water holding capacity and permanent wilting point were 24.5% and 6.5% in soil profile of 120 cm, respectively, according to the observation data from local weather station. Local geomorphic type is mainly characterized by hilly and gully. The physical and chemical properties of soil in 0–30 cm soil layer are as follows: soil organic carbon of 10.2 g kg^−1^, total soil nitrogen of 0.65 g kg^−1^, readily available phosphorus concentration of 8.7 mg kg^−1^, total salinity content of 0.324 g kg^−1^ and average pH of 5.9, respectively.

### Experimental design and field management

The study was a 3 × 3 factorial experiment in a randomized complete block design with three replicates. The three alternative ridge-furrow units differing mulching materials included 1) FT, alternative ridge-furrow mulched with transparent polyethylene film (0.008 mm thick and 1.2 m width) (Fig. 5b), 2) FB, alternative ridge-furrow mulched with black polyethylene film (0.01 mm thick and 1.2 m width) (Fig. 5c), and 3) CK, alternative ridges and furrows with no mulching as conventional planting pattern (Fig. 5d), respectively. For both introduced treatments, each ridge-furrow unit comprised two sorts of ridges in different sizes, i.e. wide but low ridge (0.6 m in width and 0.15 m in height) and narrow but high ridge (0.4 m in width and 0.3 m in height) (Fig. 5a). The ridges were used to produce and collect rainwater runoff (mainly on narrow-high ridge) and for walking and field operation (mainly on wide-low ridge). Moreover, the furrows at the junction between two ridges can be served as the zone of rainwater congestion and crop planting. The plastic film products used were all made in Lanzhou Green Garden Corporation, China. Three maize cultivars with different maturity groups consisted of, 1) KDVI, an early-maturating cultivar with growing season length of 93 days, was widely sown in this area, 2) JK3, mid-maturing variety, known as the cultivar of strong tolerance to drought environment, average growing duration is 101 days, and 3) FN1, a high-yield but late-maturing cultivar with growth duration of 108 days. Each plot was 5 m long by 5 m wide and surrounded by ridges to prevent surface water runoff.

Before sowing, all of 27 plots were located and equally split into three blocks in each season. The ridge-furrow unites in each plot were manually built up in early May 2012 and late November 2012, respectively. There was a fallowing period between after harvesting in first growing season and before planting in second growing season, during which the ridges were remained as they were. Before planting in second growing season, the ridges were *in situ* maintained or somewhat repaired if needed. Meanwhile, weeds were manually cleaned in all plots. For the FT and FB plots, the plastic film was covered by hand immediately after the completion of ridges and furrows in order to reduce soil water evaporation. Soil was pressed on the film surface in bands every 2 m to prevent wind from whirling away the plastic film. Two pieces of plastic films were jointed in the midline of the wide-narrow ridge, and the joint was fixed stably by placing soil on the top of the film. The seeds were sown in the furrows using a hole-sowing machineat a planting density of 125 plants plot^−1^ (equal to 50, 000 plants ha^−1^) with spacing of 40 cm inter plant distance along the furrows (Fig. 5a), on 14 May 2012 in long rainy season and 20 November 2012 in short rainy season, respectively. According to local farming practice in maize production, no fertilizer was applied for all the plots over two growing seasons. Throughout each growing season, manual weeding was undertaken in FT and CK plots at the stages of jointing and flowering but not in FB plots where the weeds were well controlled across two growing seasons. All the plants were hand harvested in early-September and late-February in 2012 and 2013 growing seasons, respectively. After harvesting, plastic residue was completely cleared by hand in all the plots.

### Measurements and methods

An automatic temperature logger (MicroLite-U, Fourier, USA) placed in the central between two plants in the furrows of each plot was used to record soil temperature at 10 cm depth in both growing seasons. Total soil water storage (SWS, mm) at 0‒120 cm soil layer was calculated from soil gravimetric moisture content (GSW, %). At main developmental stages such as sowing, seedling, jointing, heading, filling and maturity, soil samples at each 20 cm increments within the depth of 120 cm was collected using a 0.08‒m diameter hand auger by randomly selecting three points every plot in the central of two plants in furrows. Soil water content was determined gravimetrically for each sample. Soil water storage was calculated as follows:

1 $$SWS(mm)=GSW( \% )\times pb(g\,c{m}^{-3})\times SD({\rm{mm}})$$

where ρb was soil bulk density (1.48 g cm^−3^) and SD referred to soil depth.

### Phenology observation, leaf area and above-ground biomass

Maize BBCH-scale was used to identify the length of main developmental stages. The phenology in each plot was surveyed and the dates of beginning of seedling (defined as the date when 50% of the plants grew at least three leaves), beginning of jointing (defined as the date when 50% of the plants had first node), heading (defined as the date when tassel emerged in 50% of the plants), beginning of filling (defined as the date when kernels at blister stage with about 16% dry matter) and maturity (black dot/layer visible at base of kernels, about 60% dry matter) were recorded in both seasons. At heading stage, three plants in each plot were cut at ground level and total green leaf area were hand measured for each plant. Above-ground biomass were dried in an oven at 105 °C for 1 h and then at 75 °C for a minimum of 72 h.

At silking, six plants grown in inner rows were tagged in each plot and potential kernels per ear were determined by counting the number of spikelet for each ear. The aborted kernels per ear were also quantified according to Otegui *et al*.^51^ The kernel abortion was therefore calculated as the ratio of aborted kernels to potential kernels per ear. At maturity, twelve maize ears from each plot were randomly sampled from the inner rows for the determination on yield components, i.e. ear length, ear diameter, kernel number per ear, kernel weight per ear and thousand-grain weight. Plot grain yield was obtained by harvesting all the ears from the middle 6 rows (equal to 15 m^−2^) of plants, shelled manually and then placed all samples into forced-air oven at 105 °C for 1 h and at 80 °C for a minimum of 72 h.

### Economic water use efficiency (WUE) determination

The WUE (kg ha^−1^ mm^−1^) was calculated using the following formula:

2 $$WUE=\frac{Y}{ET}$$

where Y is grain yield (kg ha^−1^), ET (mm) is evapotranspiration in crop growing season.

The experiment was carried out under rainfed conditions and no irrigation was provided during growing season. Local rainfall during the growing season was too scarce to cause drainage below 120 cm underground. Evapotranspiration (ET, mm) was calculated using the following formula:

3 $$ET=P+{\rm{\Delta }}SWS$$

P (mm) was the total rainfall during the growing season and ∆SWS (mm) was the difference in soil water storage (0‒120 cm) between the beginning and the end of the growing season.

### Statistical analysis

There were three farming patterns including FT, FB and CK, and three maize genotypes with different maturity groups (i.e. early-, mid- and late-maturing traits) used in this study. Each combination of farming pattern and crop genotype was replicated for three times through a randomized complete block design. Using the PROC MIXED of SAS (v. 9.3; SAS Institute Inc., Cary, NC), two-way factorial analyses of variance (ANOVA) were performed separately for each growing season in order to determine the statistical significances in each of the parameters such as soil water storage, phenological duration, leaf area, above-ground biomass, yield components, grain yield and water use efficiency among farming patterns, crop genotypes and their interactions. Particularly, farming pattern, crop genotype and their interaction were assigned as fixed factors, and block was viewed as random factor. The fixed effects were calculated using Type 3 estimable functions. Fisher’s LSD test was further used to evaluate significant differences in the means of each combination between farming pattern and crop genotype when the fixed effects were found to be statistically significant in ANOVA analysis (p ≤ 0.05). The multiple comparisons were not performed once an insignificant fixed effect was detected. All statistical analyses were performed with SAS 9.3 software.

## Electronic supplementary material

Supplementary Information

## References

[1] Conway, D. In *The East African great lakes: limnology, palaeolimnology and biodiversity* (ed. Odada, E.O. & Olago, O.O.) 63–92 (Kluwer Academic Publishers, 2002).

[2] Usman MT, Reason C (2004). Dry spell frequencies and their variability over southern Africa. Clim Res..

[3] Vohland K, Barry B (2009). A review of *in situ* rainwater harvesting (RWH) practices modifying landscape functions in African drylands. Agric Ecosyst Environ..

[4] Müller C, Cramer W, Hare WL, Lotze-Campen H (2011). Climate change risks for African agriculture. Proc. Natl Acad Sci USA.

[5] Morton JF (2007). The impact of climate change on smallholder and subsistence agriculture. Proc. Natl. Acad. Sci. USA.

[6] Hatibu, N. & Mahoo, H. In Conservation tillage with animal traction. *A resource book of the Animal Traction Network for Eastern and Southern Africa* (*ATNESA*) (ed. Kaumbutho, P.G. & Simalenga, T.E.) 161–171 (Harare, 1999).

[7] Van Dijk, J. A. & Ahmed, M. H. *Opportunities for expanding water harvesting in Sub-Saharan Africa: The case of the Teras of Kassala*. No. SA40 (Gatekeeper Series, International Institute for Environment and Development, London, 1993).

[8] Mati, B. & de Lange, M. In Proceedings of the Symposium and Workshop on Water Conservation Technologies for Sustainable Dryland Agriculture in Sub-Saharan Africa (WCT), Bloem Spa Lodge and Conference Centre. 60–74 (South Africa, 2003).

[9] Malley ZJU, Kayombo B, Willcocks TJ, Mtakwa PW (2004). Ngoro: an indigenous, sustainable and profitable soil, water and nutrient conservation system in Tanzania for sloping land. Soil Till. Res..

[10] Stroosnijder, L. In Proceedings of the Symposium and Workshop on Water Conservation Technologies for Sustainable Dryland Agriculture in Sub-Saharan Africa (WCT) (ed. Beukes, D., de Villiers, M., Mkhize, A., Sally, H. & van Rensburg, L.) 92–102 (South Africa, 2003).

[11] Falkenmark, M., Fox, P., Persson, G. & Rockström, J. Water harvesting for upgrading of rainfed agriculture. *Problem Analysis and Research Needs. SIWI Report II. Stockholm International Water Institute* (Sweden, 2001).

[12] Ngigi SN (2003). What is the limit of up-scaling rainwater harvesting in a river basin?. Phys. Chem. Earth, Parts A/B/C..

[13] Aberra Y (2004). Problems of the solution: intervention into small-scale irrigation for drought proofing in the Mekele Plateau of northern Ethiopia. Geogr J..

[14] Zhou LM, Li FM, Jin SL, Song Y (2009). How two ridges and the furrow mulched with plastic film affect soil water, soil temperature and yield of maize on the semiarid Loess Plateau of China. Field Crops Res.

[15] Gan Y (2013). Ridge-furrow mulching systems-an innovative technique for boosting crop productivity in semiarid rain-fed environments. Adv Agron..

[16] Mo, F. *et al*. Development and application of micro-fieldrain-harvesting technologies. *Trans Ch Soc Agric Eng*. **29**, 1–17 (2013). (In Chinese).

[17] Wang TC, Wei L, Wang HZ, Ma SC, Ma B (2011). Responses of rainwater conservation, precipitation-use efficiency and grain yield of summer maize to a furrow-planting and straw-mulching system in northern China. Field Crops Res..

[18] Liu CA (2009). Effects of plastic film mulch and tillage on maize productivity and soil parameters. Eur J Agron..

[19] Han J, Jia ZK, Han QF, Zhang J (2013). Application of mulching materials of rainfall harvesting system for improving soil water and corn growth in northwest of China. J Integr Agric..

[20] Bu LD (2013). The effects of mulching on maize growth, yield and water use in a semi-arid region. Agric Water Manage..

[21] Xiaoli C, Pute W, Xining Z, Xiaolong R, Zhikuan J (2012). Rainfall harvesting and mulches combination for corn production in the subhumid areas prone to drought of China. J. Agron Crop Sci..

[22] Wang Y (2009). Effects of rainfall harvesting and mulching technologies on water use efficiency and crop yield in the semi-arid Loess Plateau, China. Agric Water Manage..

[23] Wang Q, Zhang E, Li F, Li F (2008). Runoff efficiency and the technique of micro-water harvesting with ridges and furrows, for potato production in semi-arid areas. Water Resour Manage..

[24] Li R, Hou X, Jia Z, Han Q, Yang B (2012). Effects of rainfall harvesting and mulching technologies on soil water, temperature, and maize yield in Loess Plateau region of China. Soil Res.

[25] Shimono H (2009). Genotypic variation in rice yield enhancement by elevated CO2 relates to growth before heading, and not to maturity group. ‎J Exp Bot.

[26] Haverkort, A. & Goudriaan, J. In *Efficiency of water use in crop systems* (ed. M. C. Heath, T. M. Hess, T. J. Hocking, D. K. L. MacKerron, & W. Stephens) **38**, 79–91 (Aspects of Applied Biology, United Kingdom, 1994).

[27] Stewart JI, Hash CT (1982). Impact of weather analysis on agricultural production and planning decisions for the semiarid areas of Kenya. J Appl Meteorol.

[28] Ramakrishna A, Tam HM, Wani SP, Long TD (2006). Effect of mulch on soil temperature, moisture, weed infestation and yield of groundnut in northern Vietnam. Field Crops Res..

[29] Zhao H (2012). Plastic film mulch for half growing-season maximized WUE and yield of potato via moisture-temperature improvement in a semi-arid agroecosystem. Agric Water Manage..

[30] Debaeke P, Aboudrare A (2004). Adaptation of crop management to water-limited environments. Eur J Agron..

[31] Subrahmaniyan K, Zhou W (2008). Soil temperature associated with degradable, non-degradable plastic and organic mulches and their effect on biomass production, enzyme activities and seed yield of winter rapeseed (Brassica napus L.). J Sustain Agric..

[32] Wang XL, Li FM, Jia Y, Shi WQ (2005). Increasing potato yields with additional water and increased soil temperature. Agric Water Manage..

[33] Jia Y, Li FM, Wang XL, Yang SM (2006). Soil water and alfalfa yields as affected by alternating ridges and furrows in rainfall harvest in a semiarid environment. Field Crops Res..

[34] Ai, X. Z., Song, J. F. & Xu, X. In Ginger: the genus Zingiber (ed. Ravindran, P. & Babu, K. N.) 241–278 (CRC Press, Boca Raton, 2005).

[35] Li R (2013). Effects on soil temperature, moisture, and maize yield of cultivation with ridge and furrow mulching in the rainfed area of the Loess Plateau, China. Agric Water Manage..

[36] Wang, F. & He, Z. In *Sustainable Potato Production: Global Case Studies* (ed. Zhong, Q. He., Robert, L. & Wayne, H.) 359–372 (Springer Netherlands, 2012).

[37] Zhao H (2014). Ridge-furrow with full plastic film mulching improves water use efficiency and tuber yields of potato in a semiarid rainfed ecosystem. Field Crops Res..

[38] Li XY, Gong JD, Gao QZ, Li FR (2001). Incorporation of ridge and furrow method of rainfall harvesting with mulching for crop production under semiarid conditions. Agric Water Manage..

[39] Luan, G., Wang, Y., Xie, X. & Shen, J. A preliminary study of the clear plastic mulch effect on potato. *J Chin Potato J*. **15**, 235–236 (2001). (In Chinese).

[40] Zhang S, Li P, Yang X, Wang Z, Chen X (2011). Effects of tillage and plastic mulch on soil water, growth and yield of spring-sown maize. Soil Till Res..

[41] Yi L, Shenjiao Y, Shiqing L, Xinping C, Fang C (2010). Growth and development of maize (Zea mays L.) in response to different field water management practices: Resource capture and use efficiency. Agr Forest Meteorol.

[42] Chakraborty D (2008). Effect of mulching on soil and plant water status, and the growth and yield of wheat (*Triticum aestivum* L.) in a semi-arid environment. Agric Water Manage..

[43] Stone P, Sorensen I, Jamieson P (1999). Effect of soil temperature on phenology, canopy development, biomass and yield of maize in a cool-temperate climate. Field Crops Res..

[44] Gao Y, Xie Y, Jiang H, Wu B, Niu J (2014). Soil water status and root distribution across the rooting zone in maize with plastic film mulching. Field Crops Res..

[45] Zhang J (2007). Effects of different planting patterns on water use and yield performance of winter wheat in the Huang-Huai-Hai plain of China. Agric Water Manage..

[46] Herrero MP, Johnson R (1980). High temperature stress and pollen viability of maize. Crop Sci..

[47] Hall AJ, F. Vilella N, Trapani N, Chimenti C (1982). The effects of water stress and genotype on the dynamics of pollen-shedding and silking in maize. Field Crops Res..

[48] Fageria, N. K., Baligar, V. C. & Clark, R. B. In *Physiology of crop production* (ed. Fageria, N. K., Baligar, V. C. & Clark, R. B.) 149–182 (Haworth Press Inc, 2006).

[49] Wen XX, Zhang DQ, Liao YC, Jia ZK, Ji SQ (2012). Effects of Water-Collecting and -Retaining Techniques on Photosynthetic Rates, Yield, and Water Use Efficiency of Millet Grown in a Semiarid Region. J Integr Agric..

[50] Kibe, J., Ochung’, H. & Macharia, P. Soils and vegetation of the ICRAF experimental farm (Machakos District) 68 (Detailed Soil Survey Report No. D 23. Kenya Soil Survey, National Agricultural Laboratories, Nairobi, 1981).

[51] Otegui ME, Andrade FH, Suero EE (1995). Growth, water use, and kernel abortion of maize subjected to drought at silking. Field Crops Res..

