# Supplementary material for: Yield-phenology relations and water use efficiency of maize (Zea mays L.) in ridge-furrow mulching system in semiarid east African Plateau
Source: Sci Rep. 2017 Jun 12;7:3260. doi: 10.1038/s41598-017-03372-x (PMC5468227; doi:10.1038/s41598-017-03372-x)
Supplement: Supplementary file 1 — Supplementary Information [file 41598_2017_3372_MOESM1_ESM.pdf]

Supplementary information

Yield-phenology relations and water use efficiency of maize (*Zea mays* L.) in ridge-furrow mulching system in semiarid east African Plateau

Fei Mo[a], Jian-Yong Wang[a], Feng-Min Li[a], Simon N. Nguluu[b], Hong-Xu Ren[c], Hong Zhou[a], Jian Zhang[a], Charles W. Kariuki[b], Patrick Gicheru[b], Levis Kavagi[d], Wesly K. Cheruiyot[a], You-Cai Xiong[a]*

[a] State Key Laboratory of Grassland Agro-ecosystems, Institute of Arid Agroecology, School of Life Sciences, Lanzhou University, Lanzhou 730000, China

[b] Kenya Agricultural and Livestock Research Organization, Kabete 14733-00800, Nairobi, Kenya

[c] The Institute of Botany, Chinese Academy of Sciences, Xiangshan, Beijing 100093, China

[d] United Nations Environment Programme, P.O. Box 47074-00100, Nairobi, Kenya

* Corresponding author. Tel / Fax: +86-931-8914500.

E-mail address: xiongyc@lzu.edu.cn (Y.C. Xiong)

Fei Mo and Jian-Yong Wang contribute equally to this work.

Supplementary Table 1

Supplementary Figures 1-4

Table S1 Basic systems data: maize farming system in Kenya.

**Parameters**	**data**
Land area in Kenya (ha)	56914000
Agricultural area in Kenya (ha)	27430000
Area harvested (ha) (2003-2013)	1843707
Yield (Hg ha^{-1}) (2003-2013)	16366
Production (tonnes) (2003-2013)	3003465
Seed (tonnes) (2003-2013)	55897
Major planting pattern	Fig. S1
Irrigation	Limited
Pesticide	Limited
Field management	Limited
	Drip irrigation (limited extension due to high fund input) (Fig. S2)
Innovative farming practices	Tera system (limited extension due to high labour input)
	Ridge-furrow mulching system (Fig. S3 and S4)

Data resource: http://faostat3.fao.org/home/E

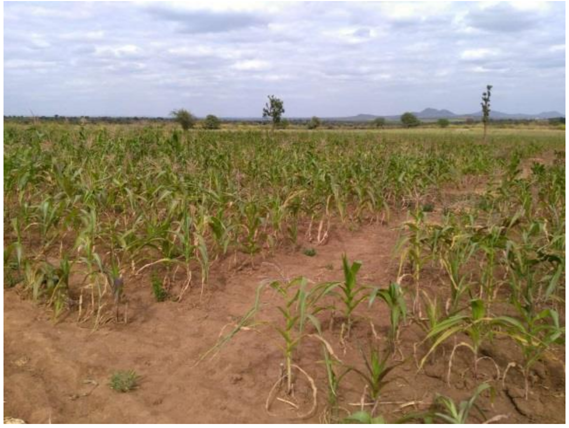

Fig. S1. Traditional flat planting

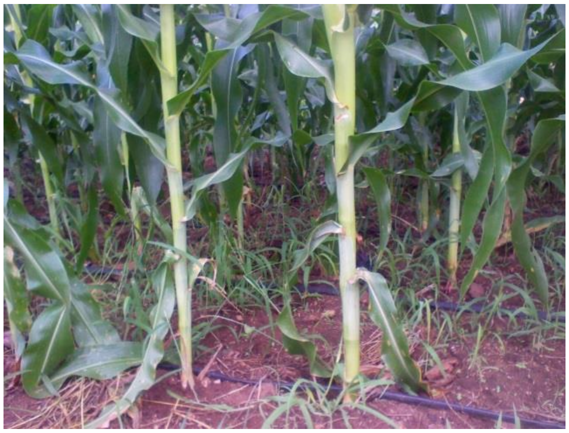

Fig. S2. Drip irrigation in maize production.

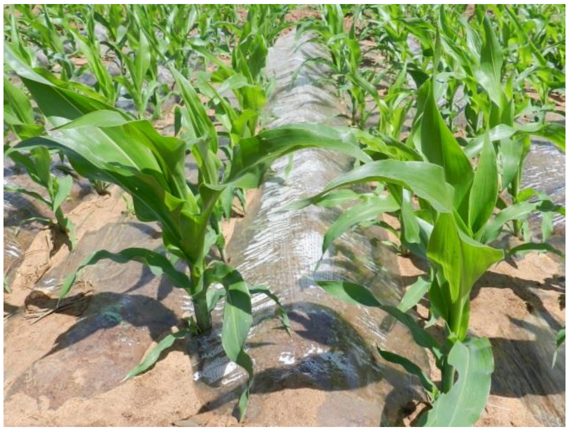

Fig. S3. Ridge-furrow mulching system.

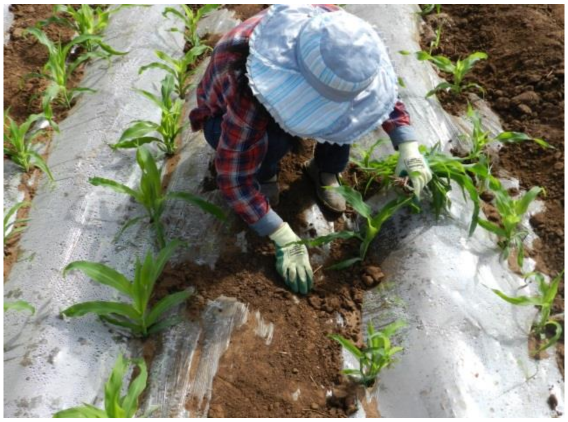

Fig. S4. Ridge-furrow mulching system.
